# Supplementary material for: The Lipid Phosphate Phosphatase Wunen Promotes Eggshell Formation and Is Essential for Fertility in Drosophila
Source: Biology (Basel). 2023 Jul 14;12(7):1003. doi: 10.3390/biology12071003 (PMC10376809; doi:10.3390/biology12071003)
Supplement: Supplementary file 1 [file biology-12-01003-s001.zip › biology-2431793-supplementary.pdf]

wun^9 / wun^{23} mothers

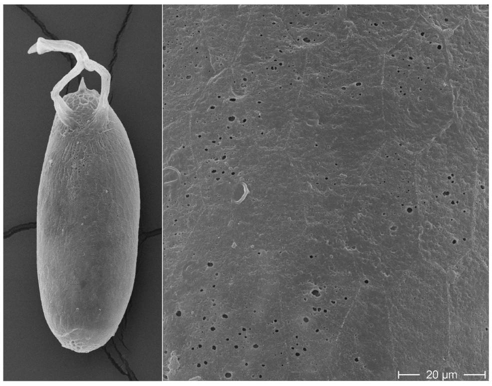

Figure S1: Eggs laid by *wun* mutant females have defective chorions.

Scanning EM micrograph of whole egg (left) and magnified portion of eggshell surface (right) laid by *wun^9/wun^{23}* mothers showing weak follicle cell impressions.
